# Protein Intake, Source and Effect on Children’s Weight Status: An Epidemiological Study in Greece

**DOI:** 10.3390/children10101606

**Published:** 2023-09-27

**Authors:** Stamatia Kokkou, Venetia Notara, Aikaterini Kanellopoulou, Areti Lagiou, Demosthenes Panagiotakos

**Affiliations:** 1 Department of Public and Community Health, Laboratory of Hygiene and Epidemiology, School of Public Health, University of West Attica, Alexandras Avenue 196, 115 21 Athens, Greece; 2 Department of Nutrition and Dietetics, School of Health Sciences and Education, Harokopio University, Thiseos 70, 176 76 Athens, Greece

**Keywords:** children, overweight/obesity, plant, animal, total, protein

## Abstract

The development of overweight and obesity during late childhood and early adolescence is one of the most critical issues in public health. Additionally, over the past few years, the consumption of protein has tended to increase in both children and adults. The present study aimed to evaluate whether the protein type, either plant- or animal-sourced, as well as the quantity consumed, could potentially have a relationship with excess body weight in children. A total of 47 primary schools were selected, and 1728 students, aged 10–12 years, were enrolled for the purposes of this study, leading to our final sample of 712. Physical measurements, such as weight and height, were measured, and children were separated into two weight status categories according to their Body Mass Index, which was obtained through the International Obesity Task Force criteria. Students’ diets were assessed through a questionnaire, and total, plant- and animal-derived protein intakes were calculated. Unadjusted analysis showed a statistically significant association between body weight and plant protein. After adjustment for overall energy intake, sex, age, and exercise, there was a statistically significant negative association between plant protein and excess body weight in children (OR: 0.964, 95% CI: 0.936; 0.992), but a lack of association for animal-sourced protein (OR: 1.002, 95% CI: 0.991; 1.013). Dietary plant-derived protein seems to have a positive effect on weight status in children, independently of total energy intake and physical activity; nevertheless, additional research is required in order to elucidate the underlying mechanisms.

## 1. Introduction

During the past few decades, mainly since the beginning of the 2000s and during the new millennium, an emerging desire to seek pathways and solutions, as well as a great effort, has been observed among medical and public health research scientists focused on lifestyle and nutrition-pivoted factors. One of those areas, surrounded by great interest within the scientific community, concerns dietary habits associated with the consumption of protein. Protein consumption is not a surprising area of interest due to the fact that consumers tend to increase their intake, compared against dietary habits from the last century and official recommendations. Specifically, recent data indicate that total protein consumption has risen globally, reaching a daily amount of 78.2 g/day, whereas mean global intakes for protein derived from animal and plant sources were 33.3 g/day and 29.1 g/day, respectively [1]. To put that into perspective, according to the latest Dietary Reference Intakes (DRIs) established by the National Academy of Medicine (United States of America), the Recommended Dietary Allowance (RDA) of protein for adults is 56 g/day for men and 46 g/day for women [2]. Furthermore, as expected, the respective RDA values for children are lower, ranging from 34 g/day for ages 9–13 and up to 52 and 46 g/day for boys and girls aged 13–18 years [2]. The aforementioned official recommendations are much lower than what was observed for adults to receive, which, as a fact, is quite worrying about the reality of children’s habits and the effect of those on their health. The apparent point of adults’ overconsumption of protein raises the question of whether children follow the same pattern or not, as well as what the ratio is between animal-based and plant-based sources in their diet. The latter could potentially present as a key indicator of overall diet quality due to the fact that each source type is accompanied by different macro- and micro-nutrient content, thus being related to varying intakes of those substances. Additionally, the difference between animal-derived and plant-derived protein consumption could be interpreted as an aspect possibly associated with body weight and the manifestation of overweight and/or obesity in childhood. More and more consumers tend to adopt dietary patterns higher in protein, possibly due to the surge in popularity of the Western diet, which is rich in this macronutrient, primarily derived from meat and processed meat products [3]. This fact is a crucial point for public health and medical science professionals due to the fact that it comes in contrast with the proposed Mediterranean diet model, which is rich in fruit, vegetables, nuts, and other plant victuals and has much lower protein content, compared with the aforementioned western type, sourced mainly from dairy products and pulses, offering overall fewer quantities of animal foods [4]. The difference regarding each dietary pattern’s dominant providing source, namely for the former animal products and the latter plant-based ones, could be a key indicator for the initial evaluation of a person’s dietary habits.

The existing literature provides data supporting both sides of the coin regarding protein consumption; it is, nonetheless, mainly focused on adult consumers. Although there is evidence supporting the beneficial effects of excess dietary protein in multiple aspects, such as weight management, appetite, and risk for cardiometabolic disease [5], there is also evidence pointing to an increased risk of developing type II diabetes mellitus in adulthood due to the consumption of more significant amounts of animal protein [6]. Furthermore, a recent study comparing high- and low-protein formula in infants concluded that the latter had a protective effect against increased body weight at 11 years, as well as a protective effect regarding adiposity, similar to the impact of breastfeeding [7]. Additionally, a very recent meta-analysis pointed out the probability of a causal relationship between greater dietary protein content, with emphasis on animal protein, and elevated body weight in childhood, noting, however, that there is a lack of relevant studies in the literature [8]. Moreover, the evidence regarding the effect of protein intake and health biomarkers related to cardiovascular diseases (CVDs), such as blood pressure and blood lipid levels, is conflicting and insufficient, according to a recent meta-analysis [9]. Nevertheless, it is essential to acknowledge that there is very limited and inconclusive evidence about the type of protein, as in plant vs. animal, and its effect on such markers [10,11,12].

Based on the findings mentioned above, the aim of the present work was to investigate overall protein intake, as well as protein source, and their potential association with weight status in a sample of Greek children aged 10 to 12 years.

## 2. Materials and Methods

### 2.1. Design

The present work constitutes the result of a population-based, observational cross-sectional study carried out in the academic years 2014–2015 and 2015–2016 in three significant regions of Greece, namely the main area of Athens, as well as Heraklion, which is the capital city of Crete Island, and three Peloponnese peninsula counties, particularly Sparta, Pyrgos, and Kalamata. The areas of which the sample was derived were selected because of their representativeness of both large urban and rural municipalities. A total of 47 primary schools were chosen using random sample selection from a list of Greek primary schools provided by the Greek Ministry of Education. Of those primary schools, 32 were located in Athens, the capital city of Greece, 5 were in Heraklion, and the remaining were in the previously mentioned big cities on the Peloponnese peninsula.

### 2.2. Participants

The overall sample of the study consisted of a total number of 1728 students (46% boys) aged 10–12 years. For the present analysis, only children with available information regarding dietary habits were included. Missing data regarding either dietary habits or anthropometric characteristics was a reason for exclusion; thus, the final sample consisted of 712 students from those five areas in Greece. No difference in the baseline characteristics (i.e., sex, age, weight status, and exercise) was found amid students included in the analysis and those excluded. The participation rate among the 47 schools extended from 95% to 100%.

### 2.3. Power Analysis

The present sample of 712 children was adequate to estimate the effect size measures’ differences between the study groups of 15% on the prevalence of overweight/obesity, reaching 83% statistical power at <5% level of significance.

### 2.4. Body Status Assessment

Students’ height and weight measurements were evaluated using standard procedures. Based on those, the Body Mass Index (BMI) was calculated, specifically as weight (measured in kg) divided by height (measured in m) squared. Furthermore, based on the sex- and age-specific BMI cut-off criteria set by the International Obesity Task Force (IOTF), children’s weight status was classified into two categories: “normal” or “overweight/obesity” [13]. Due to the fact that very few children were living with underweight, they were added to the “normal” weight status group. For the purposes of this analysis and because of the number of children living with obesity in our sample being very small, the two categories of increased body weight, “children living with overweight” and “children living with obesity” were merged into one, as explained.

### 2.5. Measurements

A self-reporting questionnaire was given to students to complete. The presence of trained personnel, specifically field researchers and teachers, aimed only to help explain any misconceptions, not to assist in answering any questions or guiding them as to what is “more correct”. The printed document included questions regarding students’ socio-demographic characteristics, such as age and sex, as well as a validated Food Frequency Questionnaire (FFQ) [14] and a validated questionnaire on physical activity [15]. Based on the former, children’s dietary habits were further analyzed by their macronutrient content (protein, carbohydrate, and lipid) with the use of the “Composition tables for Greek foods and recipes” [16] and the composition tables of the United States Department of Agriculture [17]. Macronutrient content, measured in grams, was calculated for each answer’s serving size, and, with the use of those data, daily intake was calculated. Protein content was additionally classified into two categories regarding its source, namely “animal protein” and “plant protein”. The overall evaluation of participants’ energy intake was assessed through their FFQ records [14] by calculating the caloric content of each food product according to their serving size. Participants’ exercise status, presented as “physical activity”, was defined as their engagement in extracurricular athletic activities, like daily or weekly participation in sports teams, swimming, jogging, etc., and was categorized into a binary variable (no or yes). All students’ athletic activities were recorded through a reliable and accordingly validated questionnaire created for children and adolescents (Physical Activity and Lifestyle Questionnaire (PALQ)) [15].

### 2.6. Statistical Analysis

Data presented from continuous variables are expressed as mean value ± standard deviation and from the categorical ones as absolute and relative (%) frequencies. For the evaluation and confirmation of the normality of distribution for continuous variables among the different weight status categories, namely total, animal, and plant protein, the Kolmogorov–Smirnov test was used. Concerning the consumption for each of the variables mentioned above between the two weight status groups, means were compared with the use of Student’s *t*-test. Additionally, the comparison between students’ overall protein intake and the appropriate RDA was performed using Student’s *t*-test, and the results are shown in the form of a histogram. In order to assess the impact of protein intake on the development of overweight and obesity, both simple logistic regression models, and multivariable ones, were carried out, and the results are demonstrated as odds ratios (OR) along with the corresponding 95% confidence intervals (95% CI). For the estimation of the possibility of multicollinearity amongst the independent variables, the variance inflation factor and tolerance were assessed. The analysis was based on the official DRIs for protein, which are presented as grams/day, and for the specific age study group and are the same in both genders [2]. All statistical analyses were performed with the use of Stata 14.0 (M. Psarros & Assoc., Sparti, Greece) with the level of significance set at 5%.

### 2.7. Bioethics

The present study was conducted in agreement with the Declaration of Helsinki (1989) and was further delegated by the Institute of Educational Policy as part of the Ministry of Education and Religious Affairs (code of approval F15/396/72005/C1). All those involved in this study—students and their parents, teachers and administrative staff—were thoroughly familiarized about its purposes and processes, and students’ parents and/or guardians were requested to undersign an informed consent form.

## 3. Results

The baseline characteristics of the students are shown in Table 1, both in total and separately for each weight status category. Participants had a mean age of 11.23 ± 0.787 years, and 41.6% were male. The sex of students living with overweight and/or obesity did not differ (boys: 50.5%); however, female students were most likely to belong in the normal weight category (boys: 38.4%), a result which was statistically significant (*p* = 0.004). The sample’s mean consumption for total protein, measured in grams, was 58.68 ± 33.97, whereas for animal and plant protein, it was 37.15 ± 24.73 and 21.53 ± 13.79, respectively. Regarding the latter type, mean intake was significantly greater in the children of “normal” weight (22.54 ± 14.35 vs. 18.65 ± 11.57, *p* < 0.001), though a non-significant association, though marginally, was noted for total intake (*p* = 0.062) and no significant association for animal-sourced (*p* = 0.470). Additionally, among students living with overweight/obesity, the percentage of animal protein consumed was significantly higher compared with the percentage of plant protein (*p* = 0.007). It was quite interesting to observe that mean daily energy intake was significantly decreased in students living with either overweight or obesity (*p* = 0.031). Lastly, most children were physically active (yes: 79.5%); however, children of normal body weight were significantly less likely to be inactive compared with their counterparts (*p* = 0.002).

**Figure 1:**
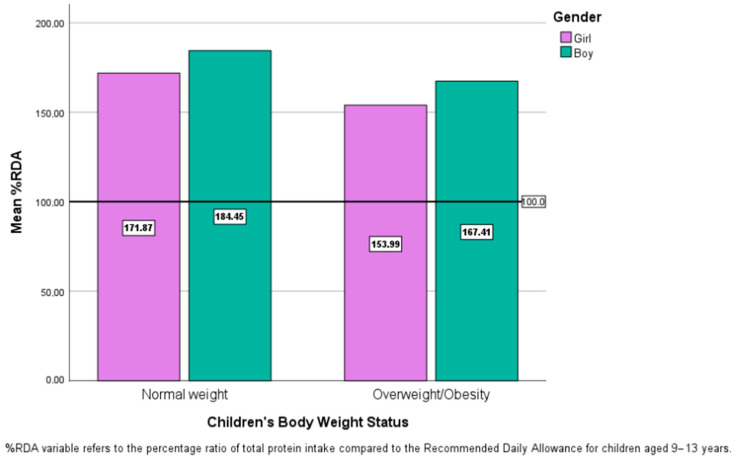
Comparison of children’s overall protein intake in relation to the Recommended Daily Allowance for their specific age group, categorized based on their weight status and sex.

The mean total protein consumption for each gender and both weight status categories, expressed as frequency relative to the RDA for children aged 9–13, is shown in Figure 1. Mean protein intake was statistically higher than the RDA for the specific population-of-interest in all groups (all *p* values < 0.05).

Logistic regression analysis, adjusted only for the overall daily energy intake, is shown in Table 2, separately for each type of protein. There was a statistically significant positive association between plant-protein intake and weight status (OR: 0.961, 95% CI: 0.935; 0.988), whereas no association was shown for animal-sourced and total protein consumption.

Table 3 presents models of multiple logistic regression for the assessment of the potential relationship between students’ weight status and plant- and animal-derived protein consumed. Model 1, which was the initial, unadjusted analysis, showed a statistically significant inverse association between plant proteins and the presence of either overweight or obesity (OR: 0.969, 95% CI: 0.952; 0.987); however, no association was found for animal protein (OR: 1.005, 95% CI: 0.997; 1.014). Those results remained when total energy intake was added as a potential confounder (Model 2), for both plant (OR: 0.963, 95% CI: 0.936; 0.990) and animal protein (OR: 1.003, 95% CI: 0.992; 1.014). The concluding model (Model 3) was additionally adjusted for sex, age and physical activity, and the statistically significant association between plant-sourced protein and body weight status remained once more (OR: 0.964, 95% CI: 0.936; 0.992), whereas additional significant inverse associations for childhood overweight/obesity were observed for girls compared with boys (OR: 1.649, 95% CI: 1.167; 2.331) and physically active children compared with the more sedentary ones (OR: 0.509, 95% CI: 0.339; 0.763).

## 4. Discussion

The present analysis tried to evaluate the potential relationship between students’ body weight status and factors regarding protein consumption, such as source and mean intake for each type (animal vs. plant). Total protein intake did not have any association with the outcome in question. In contrast, after adjustment for multiple covariates, only plant protein was observed to have a statistically significant protective effect against increased body weight. At the same time, no association was shown for animal protein intake. Moreover, total protein intake was elevated in both sexes and both weight categories, reaching up to 184.45% of the age-specific RDA, a result that was statistically significant.

The observed protective effect of plant-derived protein in early adolescence against the existence of either overweight or obesity in children’s nutrition could be attributed to its sources’ satiety-related qualities. Specifically, the primary dietary source of plant protein is legumes and pulses, as well as their derivatives [16,17], meaning food products that have been shown to improve the feeling of fullness immediately after the meal [18]. Additionally, in a more recent study comparing the satiety effect of a plant-based meal and an isocaloric and macronutrient-matched animal-derived meal, the outcome was in favor of the former rather than the latter, indicating a potentially beneficial effect on appetite and weight management-related issues [19]. After taking into consideration the result of another meta-analysis, which concluded that victuals that enhance satiety have a positive impact on weight management [20], our results could be partially explained. It is worth mentioning, though, that in a 2023 study seeking to elucidate the relationship, no association between the source of the meal, as in plant or animal-derived, and overall satiety was observed [21]. However, according to a systematic review focusing on the impact of plant-based nutrition on the accumulation of body fat, the evidence was inconclusive, a notion that was attributed to the limited data about the potential relationship in the existing literature [22]. Moreover, results from a recent randomized trial indicated a potentially favorable effect of a plant-based dietary model on both children’s weight status as well as various other cardiovascular disease risk factors, such as blood pressure and dyslipidemia indicators [23]. Finally, another study, an intervention aiming to examine the effect of a plant-based nutrition model on either overweight or obesity, observed a statistically significant positive alteration in adult participants’ body weight, but no such association was found among underage participants [24].

Furthermore, our analysis showed that animal-sourced and total protein consumption were not statistically associated with elevated body weight in children, expressed as either overweight or obesity. However, the latter was shown to range between 153.99% and 184.45% of the Recommended Dietary Allowance for our sample’s age category, which was mainly attributed to the high percentage of animal protein in children’s nutrition. Indeed, our result showed that the quantity of animal-food-derived protein intake alone was higher than the RDA when, at the same time, plant-food-derived protein intake did not cover the RDA in both weight status groups. Increased protein intake, as well as its sources, regarding issues of body development and body weight management, is a subject not yet thoroughly investigated according to the existing literature. Nevertheless, concerning body development and accumulation of adipose tissue, it is documented that higher protein intake at 12 months and 5–6 years of age was associated with increased body weight at seven years of age [25]. This is in accordance with a more recent systematic review, which associated elevated both animal-derived and total protein intake in infancy with increased body weight during both childhood and adolescence [26]. Our results suggest the existence of an association, as it seems that there is a link between the type of protein consumed earlier in life and immoderate body weight during childhood, and simultaneously, it is shown that the quantity consumed over-exceeds the official recommendations and could potentially present a part of the aforementioned observed relationship. On the other hand, regarding body weight management, data is scarce for the factors in question. Another study evaluating the effect of a normal-protein and a high-protein diet on weight loss and appetite in children/adolescents during their stay at a weight-loss camp concluded the quantity of protein in the sample’s diet did not differentiate the outcomes, as both diets achieved similar results [27]. A similar study, in the environment of a weight-loss camp, reported that two isocaloric diet models, a standard and a high-protein, effectively led to weight loss and improved body composition but with no significant difference between them and, in addition, the high-protein model did not beneficially affected appetite [28]. Finally, the observed increased consumption of protein mainly derived from animal products, even though it was not significant regarding weight status, is an issue of great interest for future research since it is indicated that children in Europe consume significant amounts of animal products [29].

The existence of some limitations should be considered when assessing the outcomes of this work. First of all, the study type is observational, and thus, there cannot be any temporal relationship or causal inferences. Second, participants originally mainly lived in urban regions in Greece; nevertheless, the study’s high representativeness was confirmed when the similarity of characteristics for both participating and non-participating regions of Greece were taken into consideration. Moreover, the size of the study sample was considerable, and thus, a stratified random sampling scheme was used, regarding school selection. Reporting bias was also limited due to the fact that trained investigators and school educators were present while the children completed the questionnaires in order to elucidate any possible misunderstanding, therefore reaffirming the validity of the responses administered by the children. Even though the data obtained from the FFQ tend to be overestimated, the specific type of questionnaire is the most commonly used in scientific research.

## 5. Conclusions

The current analysis presented a statistically significant negative association between plant-sourced protein intake and the occurrence of overweight and/or obesity in early adolescence. Children suffering from either overweight or obesity consumed lower amounts of plant-derived protein; however, no association was observed for total and animal-derived protein intake between them and their counterparts. After adjustments for potential covariates, these associations remained significant. Additionally, physical activity and gender were also significantly related to children’s weight status. Total protein consumption, that was mostly derived from animal sources, was a tremendous amount higher than the RDA in both sexes and weight status categories, a result which was also statistically significant.

To our knowledge, this is one of the first endeavors to evaluate both the type and quantity of protein consumed in an attempt to seek possible mechanisms to prevent the development of overweight and obesity in childhood and early adolescence, thus partially preventing the occurrence of associated chronic diseases, such as cardiovascular disease, later in life. It is worth commenting on the fact that lifestyle habits developed early on in life, tend to accompany us in adulthood [30], and therefore making sure those are on the healthy side, is a public health matter. This evidence suggests a great need for the elucidation of the underlying mechanisms of the observed association, as well as a need to better educate children and parents about the quality of their diet and the benefits of a more plant-based lifestyle. Nevertheless, further research is required to clarify the effect of protein intake on children and adolescents and create appropriate interventions in order to promote public health from an early age.

## Figures and Tables

**Table 1:** Characteristics of children by body weight status.

Characteristics	Overall (n = 712)	Normal Weight (n = 528)	Overweight/Obesity (n = 184)	*p*
Age (years)	11.23 ± 0.787	11.25 ± 0.786	11.17 ± 0.789	0.278
Gender				0.004 *
Boys	296 (41.6%)	203 (38.4%)	93 (50.5%)	
Girls	416 (58.4%)	325 (61.6%)	91 (49.5%)	
Protein intake (grams)				
Animal protein	37.15 ± 24.73	37.54 ± 24.53	36.01 ± 25.31	0.470
Plant protein	21.53 ± 13.79	22.54 ± 14.35	18.65 ± 11.57	<0.001 *
Total protein	58.68 ± 33.97	60.08 ± 34.25	54.66 ± 32.93	0.062
% of Animal protein	62.30 ± 12.95	61.52 ± 12.85	64.51 ± 13.01	0.007 *
Mean energy intake (kcal/day)	1392 ± 677	1424 ± 678	1299 ± 667	0.031 *
Physical activity				0.002 *
Yes	566 (79.5%)	434 (82.2%)	132 (71.7%)	
No	146 (20.5%)	94 (17.8%)	52 (28.3%)	

Data are presented for quantitative variables as mean ± standard deviation and for categorical ones as counts (percentages). Level of significance set at * *p* < 0.05.

**Table 2:** Energy-adjusted models of logistic regression for the assessment of animal protein, plant protein, and total protein on children’s weight status.

	Odds Ratio	95% Confidence Interval	*p*
Animal protein (in g)	1.006	(0.996; 1.016)	0.237
Plant protein (in g)	0.961	(0.935; 0.988)	0.004 *
Total protein (in g)	1.000	(0.990; 1.010)	0.932

Each type was separately examined through each regression model. Level of significance set at * *p* < 0.05.

**Table 3:** Multivariate logistic regression models for the assessment of the potential effect of animal-sourced and plant-sourced protein intake on students’ weight status, with adjustments for covariates.

	Model 1	*p*	Model 2	*p*	Model 3	*p*
Animal protein (g)	1.005 (0.997; 1.014)	0.237	1.003 (0.992; 1.014)	0.600	1.002 (0.991; 1.013)	0.743
Plant protein (g)	0.969 (0.952; 0.987)	<0.001 *	0.963 (0.936; 0.990)	0.009 *	0.964 (0.936; 0.992)	0.012 *
Mean energy intake (kcal)	-		1.000 (1.000; 1.001)	0.534	1.000 (1.000; 1.001)	0.548
Age (years)	-		-		0.844 (0.677; 1.052)	0.132
Sex (girl vs. boy)	-		-		1.649 (1.167; 2.331)	0.005 *
Physical activity (no vs. yes)	-		-		0.509 (0.339; 0.763)	0.001 *

Data are presented as Odds Ratio (95% Confidence Interval). Level of significance set at * *p* < 0.05.

## Data Availability

Data can be made available upon request.
